# Naïve Inflammatory Proteome Profiles of Glucocorticoid Responsive Polymyalgia Rheumatica and Rheumatic Arthritis Patients—Links to Triggers and Proteomic Manifestations

**DOI:** 10.3390/jpm14050449

**Published:** 2024-04-25

**Authors:** Allan Stensballe, Jacob Skallerup Andersen, Christopher Aboo, Anders Borg Andersen, Jie Ren, Michael Kruse Meyer, Kate Lykke Lambertsen, Peter Derek Christian Leutscher

**Affiliations:** 1 Department of Health Science and Technology, Aalborg University, Selma Lagerloefs Vej 249, 9220 Aalborg, Denmark; jacobsa@hst.aau.dk (J.S.A.); cabo@hst.aau.dk (C.A.); anders.andersen@rn.dk (A.B.A.);; 2 Clinical Cancer Research Center, Aalborg University Hospital, 9000 Aalborg, Denmark; 3 Sino-Danish Center for Education and Research, University of Chinese Academy of Sciences, Beijing 100864, China; 4 CAS Key Laboratory of Genomic and Precision Medicine, Beijing Institute of Genomics, Beijing 100101, China; renjie@big.ac.cn; 5 Department of Reumatology, North Denmark Regional Hospital, 9800 Hjoerring, Denmark; 6 Department of Neurobiology Research, Institute of Molecular Medicine, University of Southern Denmark, Campusvej 55, 5230 Odense, Denmark; klambertsen@health.sdu.dk; 7 Department of Neurology, Odense University Hospital, J.B. Winsloewsvej 4, 5000 Odense, Denmark; 8 BRIDGE, Inter-Disciplinary Guided Excellence, Department of Clinical Research, University of Southern Denmark, Campusvej 55, 5230 Odense, Denmark; 9 Centre for Clinical Research, North Denmark Regional Hospital, 9800 Hjoerring, Denmark; p.leutscher@rn.dk; 10 Department of Clinical Medicine, Aalborg University, 9220 Aalborg, Denmark

**Keywords:** polymyalgia rheumatica, rheumatoid arthritis, biomarkers, cytokines, proteomics, cell-free DNA

## Abstract

Polymyalgia rheumatica (PMR) is an inflammatory disorder of unknown etiology, sharing symptoms with giant cell arthritis (GCA) and rheumatoid arthritis (RA). The pathogenic inflammatory roots are still not well understood, and there is a lack of extensive biomarker studies to explain the disease debut and post-acute phase. This study aimed to deeply analyze the serum proteome and inflammatory response of PMR patients before and after glucocorticoid treatment. We included treatment-naïve PMR patients, collecting samples before and after 3 months of treatment. For comparison, disease-modifying antirheumatic drug (DMARD)-naïve RA patients were included and matched to healthy controls (CTL). The serum proteome was examined using label-free quantitative mass spectrometry, while inflammation levels were assessed using multiplex inflammatory cytokine and cell-free DNA assays. The serum proteomes of the four groups comprised acute phase reactants, coagulation factors, complement proteins, immunoglobulins, and apolipoproteins. Serum amyloid A (SAA1) was significantly reduced by active PMR treatment. Cell-free DNA levels in PMR and RA groups were significantly higher than in healthy controls due to acute inflammation. Complement factors had minimal changes post-treatment. The individual serum proteome in PMR patients showed over 100 abundantly variable proteins, emphasizing the systemic impact of PMR disease debut and the effect of treatment. Interleukin (IL)-6 and interferon-gamma (IFN-γ) were significantly impacted by glucocorticoid treatment. Our study defines the PMR serum proteome during glucocorticoid treatment and highlights the role of SAA1, IL-6, and IFN-γ in treatment responses. An involvement of PGLYRP2 in acute PMR could indicate a response to bacterial infection, highlighting its role in the acute phase of the immune response. The results suggest that PMR may be an aberrant response to a bacterial infection with an exacerbated IL-6 and acute phase inflammatory response and molecular attempts to limit the inflammation.

## 1. Introduction

Polymyalgia rheumatica (PMR) is a chronic inflammatory rheumatic disorder commonly occurring in people over 50 years of age and usually with an acute onset. PMR affects synovial tissue and bursa, which can lead to ongoing joint damage and irreversible disability [1,2]. Contrary to seeing it as an unexplained cause of discomfort and rigidity, imaging has shown that PMR-related symptoms arise from inflammation near the joints, such as bursitis, tenosynovitis, edema, and tendinitis, sometimes accompanied by slight joint synovitis. While the specific cause of PMR onset is still undetermined, current immunological research and clinical tests using bDMARDs (biological disease-modifying antirheumatic drugs) offer insights into the immune mechanisms at play in PMR. PMR is often associated with another rheumatic inflammatory condition known as giant cell arteritis (GCA), which can cause headaches, loss of vision, jaw pain, and scalp tenderness [3]. PMR may occur independently or with giant cell arteritis [2]. Rheumatoid arthritis (RA) is a chronic autoimmune inflammatory disease that affects synovial tissue resulting in common clinical features of RA, i.e., pain, fatigue, joint swelling, and morning stiffness [4]. Early- (ERA) and elderly-onset RA (EORA) may differ in pathogenesis and age distribution in which EORA has a different pro-inflammatory cytokine profile, including higher IL-6 and lower TNF-α, and the susceptibility of genes differs [2]. Since EORA exhibits PMR-like symptoms, it is sometimes difficult to differentiate it from PMR.

Despite PMR’s notable prevalence with a lifelong risk of PMR being 2.43% for women and 1.66% for men in the United States [5], and with the highest incidence, ranging from 41 to 113 cases per 100,000 people aged 50 years and older, in Scandinavia [6], PMR remains a poorly understood disease, especially when contrasted with the substantial body of research dedicated to RA [7]. Although PMR does not clearly impair survival or organ function, it can have a detrimental effect on quality of life, due to the subacuteness of symptoms.

Hitherto, PMR is a significant medical challenge within the realm of rheumatology having no specific diagnostic tests or unique pathological symptoms and, thus, identified based on clinical characteristics [1,8]. Notably, its overlapping clinical features with other conditions—particularly RA being the most common differential diagnosis and rheumatic disease—often complicate the diagnostic process. PMR is considered to represent a chronic systemic inflammatory pathology, characterized by new onset bilateral shoulder and hip girdle pain (which cannot be explained by an alternative condition) and stiffness with raised inflammatory markers (2012 EULAR ACR Criteria). A diagnosis primarily depends on symptoms, signs, and inflammation markers. While ultrasound, magnetic resonance imaging (MRI), and positron emission tomography–computed tomography (PET-CT) offer potential advancements for investigating suspected PMR, their limited availability, high costs, and uncertain diagnostic efficiency pose challenges. Thus, symptoms like pain and stiffness can often mimic synovitis, a typical feature of RA, thereby creating additional diagnostic uncertainty. Activation of acute phase reactants (APRs) is common due to the inflammatory nature of the disease. Interleukin-6 (IL-6) plays a pivotal role in inducing the liver to produce acute phase reactants, with IL-1, TNF-α, and interferon-gamma (IFN-γ) also contributing to this process [9]. Among diagnostic APRs, the erythrocyte sedimentation rate (ESR) and C-reactive protein (CRP) are laboratory findings used in diagnosis and follow-up [8].

PMR manifests by an escalating systemic inflammation, an indication of an overactive innate immune response that typically subsides over 2–3 years, after which the disease” burns out”. Despite the unique nature of this disease trajectory, the molecular mechanisms governing it remain largely elusive. Existing hypotheses suggest that an age-related decline in the adaptive immune system might trigger this inflammatory overreaction, with the potential contribution of genetic variations related to inflammation regulation by innate and adaptive immune mechanisms against unknown triggers [10,11]. Most people with active PMR have a remarkable acute phase response, increased production of IL-6 by circulating monocytes, and elevated serum concentrations of IL-6 [12].

Individuals with symptoms of PMR need to receive prompt medical evaluation and treatment to manage the symptoms and reduce the risk of complications, such as GCA. Treatment typically involves corticosteroids, which can dramatically improve symptoms, although the condition often requires long-term management. The treatment landscape for PMR is largely dominated by glucocorticoids, specifically prednisone, which provide quick symptom relief [1]. However, they do not alter the disease’s progression or prevent relapse, which is a significant challenge. The duration of a PMR attack can vary significantly among individuals. With treatment, symptoms typically improve within a few days, and treatment usually continues for one to two years. However, some individuals may experience longer treatment durations or require lifelong treatment with low doses of medication to prevent recurrence. Disease activity and remission are still difficult to assess because comorbidities, common in older adults, can produce mimicking symptoms. Moreover, the therapeutic options are limited for patients who show resistance to glucocorticoids or for those with recurrent disease. These gaps underline the critical need for a more in-depth exploration of the molecular underpinnings of PMR.

Our current study aimed to investigate the acute to post-glucocorticoid treatment of PMR, focusing on a detailed biomolecular examination of the immunological profiles. Prior to this study a search for proteins and biofluid biomarkers for PMR was conducted. A simple search for “polymyalgia rheumatica proteomics” returned zero hits on the Pubmed database and “polymyalgia rheumatica serum” returned nine hits within the last five years, including studies that investigated a single or a few proteins.

We employed quantitative proteomics to analyze the serum in PMR patients before and after glucocorticoid treatment. This approach enabled us to identify the molecular signatures associated with disease onset and their response to glucocorticoid therapy. We further evaluated a wide spectrum of pro-inflammatory cytokines and assessed circulating cell-free DNA (cfDNA) levels, providing insights into the extent of systemic immune activation in PMR and the potential involvement of neutrophil extracellular traps (NETs) in the acute phase and relevance in treatment.

Our comprehensive approach aims to enhance our understanding of PMR’s pathophysiology, improve differential diagnosis from rheumatic conditions like RA, and guide the development of more effective therapeutic strategies. Ultimately, our research seeks to advance our knowledge of common triggers and early PMR patho-immune mechanisms and contribute to improved patient treatment outcomes.

## 2. Materials and Methods

### 2.1. Experimental Strategy

The serum samples were investigated using three parallel approaches. Quantitative liquid chromatography with tandem mass spectrometry (LC-MS2)-based proteomics was applied to analyze the relative abundance change of the serum proteins. This technique can quantify the most abundant serum proteins, such as hemoglobin and complement proteins, but is unable to quantify cytokines, which are often present in low abundance. Hence, the multiplexed electrochemiluminescence cytokine analysis platform Mesoscale was applied to quantify ten key inflammatory-linked cytokines. Cell-free DNA (cfDNA) was analyzed due to its implication in several inflammatory diseases and its potential as a response biomarker to inflammatory conditions.

### 2.2. Patient Information, Study Design, and Clinical Characteristics

The study was approved by the ethical committee of Region North Denmark (N-20120074), and each participant gave informed oral and written consent to participate in the study. All patients at the Department of Rheumatology at the North Denmark Regional Hospital fulfilled the classification criteria for PMR [13]. Serum samples were obtained from nine acute PMR patients before treatment (acute PMR), and three months after treatment (active PMR). Patients were typically treated with a high-dose glucocorticoid (e.g., 50 mg prednisolone) and underwent dose reduction in the following weeks and months. Furthermore, 14 serum samples were obtained from treatment-naïve RA patients (RA), which were age-matched with 10 healthy control subjects (CTL). The diagnostic biochemistry measurements were obtained from the local clinical biochemistry department using accredited standard methods.

Based on previous experiments, we simulated data to determine the statistical power versus the limit to detect a certain fold change. With a test size (N) of 400 serum proteins, effect change of 1 (the patients were required to respond to treatment), and a sample size (n) of 9 patients, the simulation calculated a capacity to identify protein fold changes at 1.1 at 80% power. Pairing the PMR patients (*n* = 18), the capacity to identify fold change was 0.7 at 80% power.

### 2.3. Diagnostic Tests

The diagnostic biochemistry values in Table 1 were obtained from the local clinical biochemistry department.

### 2.4. Pro-Inflammatory Cytokine Measurements

The Mesoscale V-Plex human Pro-inflammatory Panel 1 (Mesoscale Discovery, Rockville, MD, USA) was applied to measure cytokine profiles in serum using a SECTOR Imager 6000 (Mesoscale Discovery) plate reader according to the manufacturer’s instructions and [14]. All samples were run in duplicates. The kit included the following cytokines: IFN-γ, IL-1β, IL-2, IL-4, IL-6, IL-8, IL-10, IL-12p70, IL-13, and TNF-α. Data were analyzed using MSD Discovery Workbench software and GraphPad Prism v9.5.1 (GraphPad Software Inc., Boston, MA, USA). One-way ANOVA (analysis of variance) tests were applied to determine statistically significant differences between groups. Samples with coefficient of variation (CV) above 25% were excluded.

### 2.5. Cell-Free DNA Levels in Serum

cfDNA was measured for all serum samples in duplicates with the Quant-iT PicoGreen dsDNA Assay Kit (Life Technologies, Carlsbad, CA, USA). Serum samples were thawed and centrifuged at 15,000× *g* for 15 min, diluted 1:25 in 10 mM Tris, pH 8.0 with 1 mM EDTA (TE-buffer). The Quant-iT™ PicoGreen™ dsDNA Assay Kit (ThermoFisher Scientific, Waltham, MA, USA) was used according to the manufacturer’s instruction using 96-well microplates, PP, and F-bottom black chimney well design (Sigma Aldrich, Darmstadt, Germany). Four-fold dilution series of DNA were included on all plates (1 µg/mL, 250 ng/mL, 62 ng/mL, 15.6 ng/mL, 3.9 ng/mL, 970 pg/mL, 243 pg/mL, 0 pg/mL). Samples and standards were prepared and measured in duplicates. Plates were measured on an Enspire multimode plate reader (Perkin Elmer, Waltham, MA, USA) with excitation 480 nm and emission 520 nm. To ensure higher confidence in the following analysis, all measurements were conducted in technical duplicates, and only values varying less than 5% between these were used for further analysis.

### 2.6. Mass Spectrometry-Based Proteomics

The preparation of serum samples was based on filter-aided sample preparation (FASP) for MS, as previously described [15]. In total, 100 µg total serum protein was prepared and digested for MS, as previously described [15]. In brief, 100 µg total protein was dissolved in 5% sodium deoxycholate in 50 mM triethylammonium bicarbonate (pH 8.6). Each sample was then reduced and alkylated using 10 mM Tris(2-carboxyethyl)phosphine hydrochloride and 25 mM chloroacetamide for 30 min at ambient temperature. Next, the samples were incubated overnight at 37 °C with 1:100 (*w*/*w*) sequencing grade trypsin (Promega, Madison, WI, USA) per sample. The peptides were extracted by solvent phase transfer, as described in [16], and stored at –80 °C until analysis.

The individual serum samples were separated and sequenced using nanoflow liquid chromatography with tandem MS (LC-MS/MS) in technical duplicates. In brief, a UPLC-nanoESI MS/MS setup with a Dionex RSLC nanopump (Dionex/Thermo Scientific, Waltham, MA, USA) was connected to timsTOF PRO mass spectrometer (Bruker Daltronics, Bremen, Germany). The peptide material was separated using a C18 reversed-phase column (Ionoptiks, 1.9 μm, 25 cm), kept at 60 °C, and eluted using a step gradient of 35 min at 350 nL per minute. A data-dependent (DDA)-PASEF MS mode with 10 PASEF scans was used. A PASEF data acquisition in the mass range of *m*/*z* 100 to 1700 was acquired at a resolution of 45,000 using ion mobility separation (IMS). The ion mobility range was set as 0.6–1.6 Vs cm^−2^ with 100 ms of ramp time and accumulation time.

### 2.7. Proteomics Data Processing and Protein Informatics Investigation

MS raw files were processed with MaxQuant, and label-free quantification was performed using the MaxQuant label-free-quantification (MaxLFQ) algorithm. Proteins with statistically significant abundance change between treatments or disease groups were identified for each group using permutation-based two-sample *t*-tests between groups. For example, polymyalgia patients before and after treatment were analyzed using a permutation-based false discovery rate (FDR) with standard parameters (FDR = 0.05, S0 = 1, and 250 randomizations). Methionine oxidation (M), protein deamination (N), and protein citrullination (R) were assigned as variable modifications.

The quantitative data were further processed in Perseus. The technical replicates were combined by taking the mean, and the missing values were imputed with random values drawn from a normal distribution to simulate signals from low-abundant proteins using standard parameters in Perseus. The resulting data table was imported into R (v 3.6.1) using PerseusR (v 0.3.4) in Rstudio (v 1.2.5001). To identify features with the highest predictive impact to identify the four patient groups, a sparse partial least squares discriminant analysis (sPLS-DA) was performed using the MixOmics package version 6.8.5. The sPLS-DA is a supervised method that identifies the most discriminative variables for classifying the plasma proteomes according to the patient group. The optimal number of components and variables on each component was found by evaluating the performance of the PLS-DA model using the MixOmics perf and tune.splsda functions with a five-fold cross-validation and 50 repetitions. Finally, a clustered image map based on hierarchical clustering was created to show the correlations between the most important variables in the sPLS-DA model and patient groups. Online informatics tools were used to investigate, e.g., gene ontology by Metascape [17], Panther [18], STRING [19], or Reactome [20].

## 3. Results

### 3.1. Patient Population and Treatment Response

The patient population data and clinical values are provided in Table 1. The PMR diagnosis satisfied the American College of Rheumatology (ACR) 1987 criteria [21]. Additional inclusion criteria were elevated C-reactive protein (CRP), sedimentation rate, ultrasound-verified synovitis [22], no cancer-related findings on an abdominal (CT), and chest X-ray to increase specificity as described by the ACR 2012 classification criteria [13]. Accordingly, the acute PMR (at enrollment baseline) patients had a high disease activity. At the three-month follow-up (termed active PMR), the patients had obtained significant effects from the glucocorticoid treatment evaluated by physical examinations and clinical biochemistry values (Table 1). The duration and disease trajectory after three months are not included in this study; however, most patients in PMR treatment continue for a minimum of 1–2 years to prevent relapse and manage symptoms. The individual dose is gradually reduced based on symptom control and ESR/CRP levels. Treatment-naïve elderly RA patients were recruited as part of the Region North Denmark patient visitation system and diagnosed by, e.g., positive anti-citrullinated protein antibody (ACPA) and immunoglobulin-M rheumatoid factor (IgM-RF). Age-matched controls were recruited as a non-inflammation background group.

### 3.2. Proteomics Profiling of Serum Identifies Normalization of PMR-Induced Inflammation

**Figure 1:**
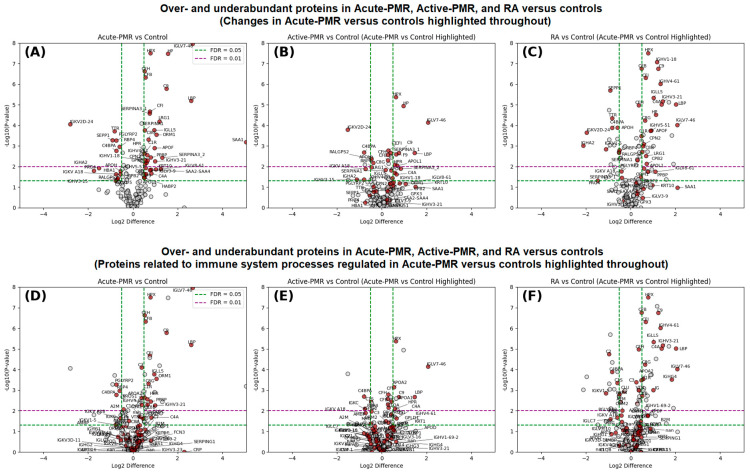
Quantitative proteomic comparisons by volcano plots and the impact on acute PMR responsive proteins and proteins associated with ‘regulation of inflammatory response’. Over- (volcano right side) and underabundance (volcano left side) illustrate the proteomic profiles of proteins with differing abundance levels in acute PMR, active PMR, and RA groups compared with a healthy control group (CTL). (**Upper panel**) Plot (**A**) highlights proteins, marked with red, that are either over- or underabundant in the comparison between the acute PMR group and CTL. Plot (**B**) highlights the same proteins in the comparison of active PMR and CTL. These proteins provide an insight into the baseline dysregulation of proteins and their subsequent changes following glucocorticoid treatment. Plot (**C**) shows the proteomic profile observed in RA patients compared with CTL with the same highlighted proteins. (**Lower panel**). In Plots (**D**–**F**), identical plots are investigated with the GO (gene ontology) biological process ’regulation of inflammatory response’ highlighted with red to showcase the inflammatory processes. Curves indicate *p*-values with FDR (false discovery rate) correction (dotted lines: FDR > 0.01; *p* > 0.05).

First, we investigated the inflammatory profiles of the cohort using quantitative proteome analysis. A total of 81 LC-MS/MS runs of serum lysates in technical duplicates and triplicates (24 CTL; 18 acute PMR; 15 active PMR; 24 RA) were analyzed by quantitative proteomics. This allowed for the identification of 349 protein groups by at least one unique peptide and 182 proteins by at least two unique high-quality peptides, at 1% permutation-based FDR and inclusion criteria of at least 50% occurrence in at least one of the four main groups (Supplementary Materials Table S2). We further correlated the quantifiable proteins between each of the three patient groups to the control group that functioned as a non-inflammatory control baseline (Figure 1A–C). Statistical analysis by two-sample *t*-tests with FDR correction between the PMR patients before and after treatment and naïve high inflammation RA compared with CTL were plotted as volcano plots (Figure 1). By comparing the shared core serum proteome of acute PMR as a baseline and three-month follow-up (active PMR) and RA, classical blood proteins, such as acute phase reactants, apolipoproteins, coagulation and complement factors, immunoglobins, and metabolic enzymes, were identified. A range of inflammation-associated proteins within the three patient groups was observed to be up- or downregulated in abundance and, in particular, was observed in the acute PMR and naïve RA groups. When comparing baseline acute PMR to the active PMR at the three-month follow-up, a normalization of inflammation levels was observed (Figure 1B). We next investigated the dynamics of protein functions in biological “regulation of inflammatory response” processes to investigate the inflammation levels between the three disease groups (Figure 1D–F). Multiple proteins were differentially abundant between the acute PMR patients and matched CTL, as seen in Figure 1D,F, with a normalization of the inflammatory condition after steroid treatment. These included several complement factors, which were more abundant in acute PMR patients.

Acute phase reactants including acute phase serum amyloid A (SAA1), complement factor 9 (C9), and lipopolysaccharide-binding protein (LBP) were consistently less abundant after three months of treatment. In contrast, plasma kallikrein (KLKB1) and hemoglobin subunit alpha (HBA1) were more abundant, and haptoglobin (HP) and leucine-rich alpha-2-glycoprotein (LRG1) were less abundant. CRP appeared to be non-regulated in all three groups compared with the CTL group, but this is probably due to non-detectable quantities in the PMR-treated patients by MS compared with CRP levels measured at the biochemistry laboratory (Table 1).

**Figure 2:**
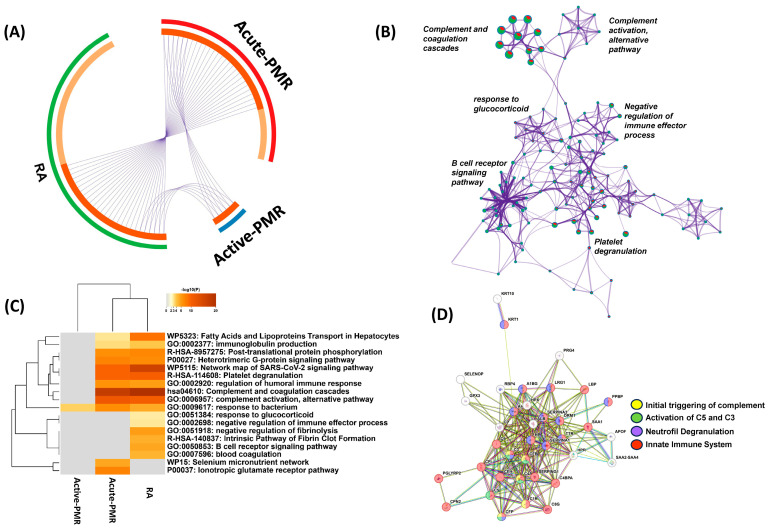
Molecular function and connectivity between over- and underabundant proteins in acute PMR, active PMR, and RA in relation to the healthy control group. (**A**) Over- and underabundant proteins connected by purple lines indicate a large overlap of identical gene products (dark orange arc: proteins in common). (**B**) Network of and size of enriched molecular terms in primarily acute PMR (green), active PMR (blue), and RA (red). (**C**) Heatmap of enriched terms across input gene lists, colored by *p*-values. (**D**) STRING analysis of over- and underabundant proteins in acute PMR and active PMR.

Next, we aimed to identify the molecular pathways associated with the initial acute phase of PMR and the proteins affected by steroid treatment. The associated over- and underabundant proteins overlapped to a high degree between the two inflammatory patient groups, acute PMR and naïve RA, while the active PMR at the three-month follow-up was associated with a far lower number of inflammatory proteins elevated from a healthy control state (Figure 1B,E and Figure 2A). Common proteins between the three groups are IGLV7-46, LBP, HP, C9, HPX, C4BPA, IGKV2D-24, and IGKV2-24. Proteins SAA1, SERPINA3, KRT10, ORM1, IGLV3-9, SAA2-SAA4, HPR, GPX3, SERPINA1, C3, and PGLYRP2 were associated with acute PMR only and not represented in the RA protein group. Furthermore, when associating the relevant proteins with gene ontology (GO)-based molecular functions, both a range of similarities and a range of differences are evident (Figure 2B–D). Most evident is the elevated level of acute phase reactants and inflammation-related proteins (Supplementary Materials Table S5). The inflammatory conditions of acute PMR and RA were dominated by active complement and coagulation-associated pathways (*p*-value 10^−31^), response to glucocorticoid (*p*-value 0.00162), and regulation of immune system-related processes (5.4 × 10^−8^), including neutrophil degranulation (Figure 2B,C). The inflammatory profile of active PMR is observed to be reduced to the basal normal state with only a slight overrepresentation of the innate immune system (GO response to bacteria). Investigating the heatmap of the common and unique GO pathways, both common and distinct molecular functions are observed in acute PMR and RA. Finally, we compared the common proteins related to acute PMR and active PMR, showing a tight functional relation among the regulated proteins in the same molecular pathways (Figure 2D).

**Figure 3:**
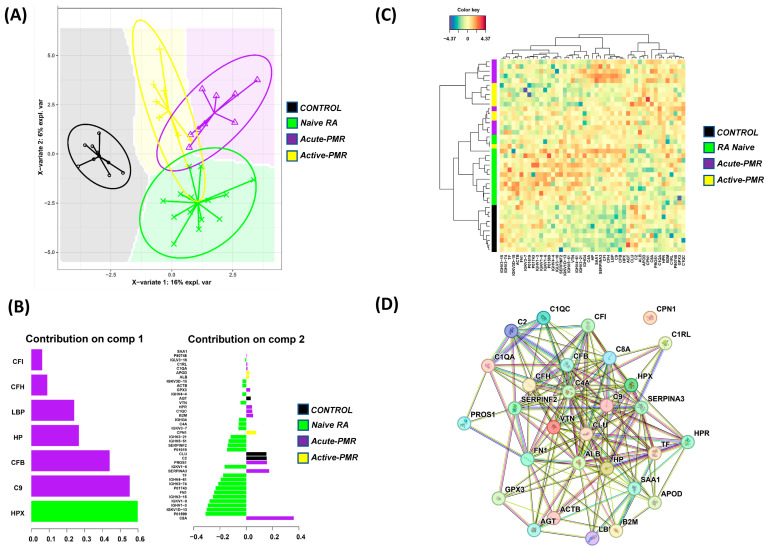
Supervised correlation analysis of the serum proteomes for discriminating patient groups and control groups. (**A**) Sparse PLS-DA score plots highlight the within-group similarities (encircled) and background prediction areas based on permutations from the first two sPLS-DA components. (**B**) The most important variables (gene identifiers) for discriminating patient groups. The bar color specifies which patient group has the maximal expression level of the individual protein. (**C**) A heatmap of the two components of the sPLS-DA shows the grouping of each patient into several clusters. The overall PMR and RA grouped together in several clusters, while the controls grouped exclusively together. (**D**) STRING analysis of protein variables associated with the discrimination of PMR patient groups.

Our next research question was to define discriminative molecular markers for the four groups based on their outcome category (acute PMR, active PMR, RA, and CTL) and, in particular, biomarkers related to acute PMR. Here, we applied a supervised sparse partial least squares discriminant analysis (sPLS-DA) to compare the overall serum proteomes between each group. The sPLS-DA analysis was able to discriminate CTL and RA from the other groups as indicated by the sPLS-DA score plot and, to a lesser degree, was able to discriminate acute PMR and active PMR from each other (Figure 3A). The most important variables for driving such discrimination on component one (C9, LBP, CFH, CFI, HPX, HP) and component two (47 proteins) between patient groups with acute PMR and active PMR are shown in Figure 3B (Supplementary Materials Table S6). These important variables were extracted and their correlation with each patient group was shown in two clustered image maps using the selected variables from both components and component one only (Figure 3C,D).

The CTL and RA patients were mainly clustered according to their groups; however, the active PMR tended to cluster with the acute PMR group (Figure 3C). When using the seven variables from component one only, the CTL patients were still clustered according to their group, and active PMR patients tended to cluster closer to the CTL patients, which could be an indication of effective treatment. A clear distinction between CTL and RA can be observed, as well as, to a lesser degree, a separation between acute PMR and active PMR. The acute PMR- and active PMR-associated inflammatory proteins were further investigated using GO and pathway correlation using STRING (Figure 3D).

### 3.3. Profiling of Pro-Inflammatory Cytokines by Multiplex Measurements and Cell-Free DNA

**Figure 4:**
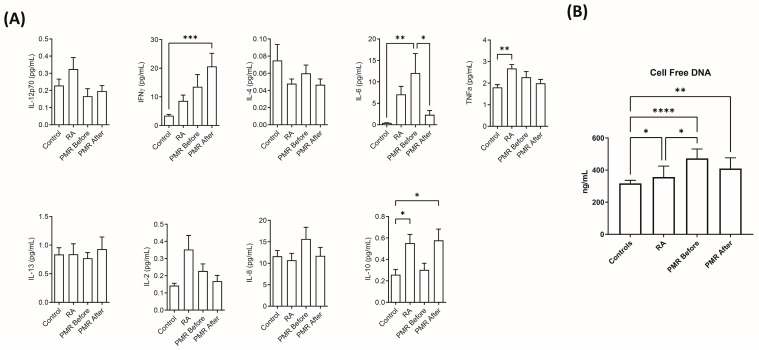
Quantitative profiling of cytokines and cfDNA: (**A**) Absolute quantification of pro-inflammatory cytokines (* *p* < 0.05, ** *p* < 0.01, *** *p* < 0.001, **** *p* < 0.0001). (**B**) The absolute concentration of circulating cell-free DNA in serum. Cell-free DNA concentrations of acute PMR, active PMR, and RA were all statistically significantly more abundant, respectively. In addition, cfDNA concentration was significantly more abundant in acute PMR before treatment compared with RA.

A multiplex panel of ten cytokines and chemokines (IFN-γ, IL-1β, IL-2, IL-4, IL-6, IL-8, IL-10, IL-12p70, IL-13, and TNF-α) were absolutely quantified by multiplex analysis (Figure 4A). We analyzed for quality and poor samples were discarded, i.e., CV > 25%. The pro-inflammatory cytokines IL6 and IFN-γ were responsive to treatment in opposite directions for the PMR patients. While IL6 was significantly less abundant (dropping by ~5.4 pg/mL), IFN-γ increased significantly after treatment. TNF-α was more abundant in the RA patients compared with the PMR patients after treatment. IL10 was not significantly more abundant in the active PMR patients after treatment while IL4 was similar between the groups. The panel of ten pro-inflammatory cytokines, except for IL-1β, was reproducibly measured and non-parametric statistics were applied as shown in Figure 4A. IL-1β may be elusive in multiplex analysis due to lability and low concentration.

The absolute concentrations of cfDNA and their median results from the cfDNA assay are given in Table 1. The concentration of cfDNA was statistically significantly higher in PMR patients before and after treatment compared with the CTLs, as shown in Figure 4B. cfDNA concentrations of acute PMR, active PMR, and RA were all significantly more abundant compared with the CTLs. In addition, cfDNA concentrations were significantly more abundant in the acute PMR treatment compared with RA. The concentration of cfDNA between acute PMR and after steroid treatment was reduced in active PMR, though not statistically significant.

## 4. Discussion

Our understanding of the immune pathophysiology of early PMR primarily originates from studies of whole blood samples [11,23,24]. In our study, we investigated and compared the serum proteome of PMR patients before and after glucocorticoid treatment with that of treatment-naïve RA and healthy CTLs. PMR and GCA are closely linked inflammatory disorders that almost always occur in people older than age 50. However, differential diagnosis with RA may occur.

To the best of our knowledge, this combined proteomic dataset represents the most comprehensive serum proteome analysis of PMR to date. Assessment of the inflammatory state of acute PMR includes a comparison with naïve RA patients before any DMARD treatment. The inclusion of PMR and naïve RA patients in this study was carefully selected to aid high specificity of the diagnosis at high disease activity. The latter patient group was selected as a proteotypic acute inflammatory disease state. We applied a combined discovery proteomics and cytokine profiling approach to investigate the serum proteome of PMR in depth. Serum contains a wide dynamic range (up to 10^10^) of proteins, which can be grouped into classical serum proteins (normal concentration range 10–50 mg/mL), tissue and cellular leakage proteins, and cytokines and interleukins (normal range 0–5 pg/mL) [25,26], and the depth of this serum proteome seems to include the first two groups. Though this is not near the number of proteins identified in, e.g., RA colon or lung tissue [27,28], it is on par with recent findings by others in terms of the total number of identified proteins in serum [29,30,31]. We did not apply any high-abundance depletion strategy because it could alter the overall proteome content. Our data suggest this when we compared our findings with Ortea et.al., who applied six protein depletion strategies [29,31].

Our applied strategy enables comparison between single patients (before and after treatment) or groups of patients to identify expression fold changes of proteins controlled by false discovery rate assessment and multiple hypothesis testing [32]. A limitation of this undepleted proteomics strategy is the missing values of undetected proteins that are not present in the entire dataset when comparing multiple samples or groups. For example, this was apparent for CRP, which was significantly reduced when looking at the serology in Table 1. This was not observed in the MS data when comparing PMR before versus after because it was below the detection range in the latter group. However, when comparing the acute PMR patients versus the naïve RA, CRP was more abundant in the PMR patients. It is, therefore, a limiting factor that proteins that are below detection in a certain group do not reach statistical significance though they could be valid biomarkers. After three months of treatment, all enrolled PMR patients were low in disease activity and all had responded well to glucocorticoid treatment. The relatively stringent inclusion criteria for the patients and treatment response resulted in a relatively high screen failure due to the same constraints in baseline and remission criteria. The patients were similar in age and CRP levels to what we observed in other larger studies [33]. This study was designed to create two paired sample groups with high systemic inflammation (before treatment) versus low inflammation (after treatment), which would be evident in the results.

The etiology of PMR is unknown but leads to a high inflammatory response, which could be perceived by the immune system to be an ongoing infection. A total of eleven proteins were overabundant in acute PMR compared with naïve RA and affected in the patient treatment (SAA1, SERPINA3, KRT10, ORM1, IGLV3-9, SAA2-SAA4; SAA4, HPR, GPX3, SERPINA1, C3, PGLYRP2). The SAA1-4 protein family, part of the serum amyloid A proteins, plays a critical role in the acute phase response to inflammation [34]. They play a crucial role in the immune response by recruiting immune cells to inflammatory sites and influencing the formation of extracellular matrix. These proteins can contribute to the recruitment of immune cells to inflammatory sites, influencing the chronic inflammation seen in PMR and serving as a marker for disease activity. SERPINA3, also known as alpha-1-antichymotrypsin, is an acute phase protein with anti-inflammatory properties. It inhibits proteolytic enzymes such as cathepsin G and chymase, which contribute to tissue damage during inflammation. Its elevated levels in PMR could be a protective response against tissue damage from inflammation. It can bind to various drugs, affecting their distribution and efficacy. In the context of PMR, ORM1 may modulate immune responses and inflammation through its effects on cytokines and interaction with immune cells. GPX3 is an antioxidant enzyme that reduces hydrogen peroxide, protecting cells from oxidative stress. Its anti-inflammatory role is crucial in neutralizing oxidative stress, a component of chronic inflammation in diseases like PMR. HPR binds free hemoglobin, preventing oxidative damage. Its role in inflammation involves protecting tissues from damage caused by the iron in free hemoglobin. In PMR, elevated HPR levels might reflect a response to prevent oxidative stress associated with inflammation. Also known as alpha-1-antitrypsin, SERPINA1 inhibits various proteases, protecting tissues from enzymes released by inflammatory cells. Its deficiency is associated with chronic obstructive pulmonary disease (COPD) and liver disease, and its role in PMR could relate to protecting tissues from inflammation-induced damage. C3 plays a central role in the activation of the complement system, an essential part of innate immunity. It facilitates opsonization, inflammation, and membrane attack complex formation. In PMR, C3 may contribute to the inflammatory cascade leading to symptoms. Last, PGLYRP2 recognizes bacterial peptidoglycan, playing a role in innate immunity by modulating the immune response to bacterial infections [35]. In addition to its role in promoting inflammation in response to bacterial infections, PGLYRP2 has anti-inflammatory properties. The involvement of PGLYRP2 in bacterial infection is multifaceted. It does not only act as a direct antibacterial agent but also plays a role in regulating the immune system’s response to ensure it is adequate and not excessively damaging to the host. Its involvement in acute PMR highlights the potential role of microbial triggers in the disease’s pathogenesis or the immune response to such triggers. This could also explain the increase in C9, LBP, and SAA1, which decreased after treatment of PMR (Figure 1A–F). Intriguingly, an autoantibody against PGLYRP2 has been identified as a promising biomarker in RA, especially in early and seronegative patients [36]. The SAA1 protein was more abundant before PMR treatment and, supposedly, has several functions, including acute phase response and chemoattractant activity. SAA1 is elevated in GCA patients compared with a small number (n = 5) of healthy controls (with no demographics reported) [37]. LBP binds lipopolysaccharides, which are part of the Gram-negative bacteria cell wall, activates toll-like receptor 4 (TLR4), and induces co-expression of CD14 in human monocytes [38]. Soluble CD14 (sCD14) has been suggested as a serum [39] and urine biomarker of RA activity [40]. Though CD14 was only reduced by 20% (not significant) after PMR treatment, the PMR patients had a 146% higher relative abundance of CD14 before treatment versus the control group (Supplemental Materials Table S2). Others propose that PMR could be triggered by a bacterial response, resulting in an aberrant immune reaction and inducing an autoantibody response to ferritin, which disappears after treatment [41]. This could explain the decrease in LBP and CD14 after treatment, which are important parts of the first line of defense against bacteria in concert with TLR4 [42]. The DMARD naïve RA patients were significantly different from the healthy controls (Figure 1C,F; Supplementary Materials Table S2). Especially, the complement system proteins were more abundant in the RA patients versus controls. In addition, C9, LBP, and SAA1 were more abundant in RA versus controls. The proteins were more abundant in the acute PMR and after three months of treatment versus RA patients, but not statistically significant (Figure 1A–C; Supplementary Materials Table S2), which suggests that SAA1 is highly elevated in PMR.

In investigating the gene ontology affected by the steroid treatment, the key molecular pathways include active complement and coagulation-associated processes, response to glucocorticoid, and regulation of immune system-related processes, including neutrophil degranulation [43]. The complement system in PMR might be abnormally activated, contributing to inflammation and tissue damage. However, in PMR, there is no well-established role for complement system dysregulation. PMR is typically associated with elevated markers of inflammation such as the CRP and ESR, but these are not specific to the complement system and elevation can be caused by other factors. A recurring observation in PMR is the heightened serum levels of IL-6. Research from two studies has indicated a rise in T17 helper cells (specifically, IL-17-producing CD4+ T cells) in the blood of PMR patients. During the active phase of the disease, B cells seem to migrate from the bloodstream into tissues, but they circulate again during remission. Many of these recirculating B cells seem to generate IL-6, aligning with their increased ability to evolve into IL-6-producing B cells when the disease is active. There are also indications of growth in pro-inflammatory T1 helper cells (meaning IFN-γ producing CD4+ T cells) and aging T cells (specifically, CD28 negative T cells) in the blood, though these observations require more extensive validation. Additionally, some RCT studies show promising results in targeting IL-6 as a treatment for PMR. For example, several studies have discussed the efficacy and safety of IL-6 inhibitors like tocilizumab in conditions such as RA, both as monotherapy and in combination with other treatments [44]. These studies suggest that IL-6 targeting could be promising in treating immuno-inflammatory rheumatic diseases, but specific data on PMR are still limited.

The etiopathogenesis of PMR is still debated. Human leucocyte antigens (HLA) and some cytokines, particularly IL-6, have been investigated where the role of triggers is hazier [45]. The top three investigated proteins are IL6, IL-1α, and TNF-α [46], but the positive correlation of the latter two is being contested [12]. The low abundant cytokines are, however, often well below the limit of detection by discovery MS; therefore, we applied the multiplex cytokine assay. The cytokine analysis revealed an interesting insight into the cytokine response to PMR treatment. The 10-plex assay roughly covers the Th1, Th2, and Th17 response and markers of inflammatory activity; therefore, it is suitable for investigating treatment response. It was expected that IL6 would decrease because of treatment, and this was confirmed, highlighting the pathogenic role of this pro-inflammatory cytokine. A recent study suggests that the humanized recombinant monoclonal antibody tocilizumab, targeting the interleukin 6 receptor (IL-6R), may be useful to treat PMR [47,48] and has since been approved for the treatment of giant cell arthritis. Several clinical trials are ongoing in RA, focusing on several targets within the IL6/IL6R and JAK/STAT pathways [49], and these could be of interest to treat PMR. Anti-IL6R trials have suggested that blockage of the IL6-IL6R-JAK-STAT signaling pathway could be viable as a treatment option [50,51]. This gives rise to JAK inhibitors as a potential treatment option, which could be explored and could provide insight into the disease. The latter drug also targets the increase in IFN-γ after PMR treatment, which was observed in our patients. The persistent Th1 response has been studied in giant cell arteritis and Takayasu arteritis (reviewed by [52]) in patients with efficient immunosuppression. Hence, JAK1 (IFN-γ) and/or JAK3 (IL6) inhibition could possibly counteract the aberrant Th1 response. IL10 increased after treatment, though not significantly. The IL10 concentration in RA and PMR patients after treatment was similar, while the IL10 levels in PMR before treatment were comparable to those in the control group (Figure 4A). This suggests that even though IL10 levels increase after PMR treatment, it is still not sufficient for the resolution of the inflammatory response.

Cell-free DNA has been suggested to be an acute phase reactant associated with coagulation and NET formation [53], and the response to treatment, though nonsignificant, of the PMR patients in this study suggests a relation to systemic inflammation. Previous studies have concluded that cfDNA could be a biomarker for early RA because the levels are significantly lower in established RA patients who receive treatment for the disease [54]. However, based on our findings, it seems that cfDNA levels are not unique to RA but are more likely linked to the inflammatory response as an acute phase marker. Our findings show an even more elevated cfDNA level associated with both naïve PMR patients and after successful steroid treatment. Furthermore, extracellular vesicles play a crucial role as mediators of neutrophil crosstalk and could be investigated concerning PMR [55,56].

Among the study limitations that may affect the presented data in our study is a higher mean age of the included PMR patients (median 70 years) compared with age-matched naïve RA (median 58 years) and healthy controls (median 52 years). The higher mean age of the PMR patients included in the study might mean these patients exhibit different disease characteristics or progression than typically observed in older populations, who are more commonly affected by PMR. This age discrepancy can lead to a misrepresentation of the disease’s typical clinical picture, potentially skewing the findings.

## 5. Conclusions

The pathogenesis of PMR is not fully understood, but it is believed to involve complex interactions between genetic predispositions, immune system dysregulation, and environmental factors. The serum response proteome of patients with PMR in glucocorticoid treatment was established and investigated. Among the proteins overrepresented in acute PMR were acute phase response proteins commonly associated with inflammation. The treatment-naïve and after-treatment results were compared with treatment-naïve RA patients and healthy controls matched to the RA patients. The most predominant cytokine response to glucocorticoid treatment was observed for IL-6 and IFN-γ, which revealed a reduction in IL6 but an increase in IFN-γ after glucocorticoid treatment. cfDNA was not significantly affected by glucocorticoid treatment of the PMR patients but was differentially abundant between the groups. Serum acute phase amyloid A was significantly less abundant in PMR patients during glucocorticoid treatment. Further research into PGLYRP2 and its functions in PMR could uncover its potential as a target for therapeutic interventions in bacterial infections and inflammatory diseases, with a focus on enhancing its bactericidal activities or modulating its impact on the immune response. Using a supervised statistical approach, we found serum proteins associated with treatment outcomes. These proteins could serve as potential biomarkers or contributors to the inflammatory processes observed in PMR and disease insight.

## Figures and Tables

**Table 1:** Patient details and clinical parameters. The characteristics of the four groups: naïve PMR patients before treatment (acute PMR), the same PMR patients during treatment (active PMR), naïve rheumatoid arthritis patients (RA), and healthy control (CTL) subjects. CRP (C-reactive protein).

Measure/Unit (Reference Values for Healthy People)	PMR Pretreatment ^1^	PMR Active Treatment ^1^	RA ^1^	Controls ^1^
Population	*n* = 9	*n* = 9	*n* = 14	*n* = 10
Age (years)	70 (10)		58 (15)	52 (12)
Ultrasound verified synovitis	9			
CT abdomen negative	9			
Chest X-ray negative	9			
CRP; mg/L (<8)	45 (27)	0 (6)	11 (31)	
Erythrocyte sedimentation rate; mm (<20)	40 (11)	6 (7)	17 (29)	
Hemoglobin; mmol/L (7.3–9.5)	7.8 (0.69)	8.6 (0.65)	8.3 (1)	
Thrombocytes; ×10^9^ (165–400)	303 (121)	286 (62)	385 (140)	
Leucocytes; ×10^9^ (3.5–10.0)	8.3 (3.74)	9.8 (1.46)	9.9 (1.75)	
Neutrophils; ×10^9^/L (2.00–7.00)	5.18 (2.43)	7.3 (1.63)	7.03 (1.68)	
Lymphocytes; ×10^9^/L (1.30–3.350)	2.59 (0.61)	1.74 (0.51)	2.16 (0.48)	
Monocytes; ×10^9^/L (0.2–0.7)	0.59 (0.20)	0.43 (0.05)	0.5 (0.22)	
Eosinophils; ×10^9^/L (<0.50)	0.18 (0.07)	0.05 (0.07)	0.12 (0.12)	
Basophils; ×10^9^/L (<0.10)	0.04 (0.02)	0.03 (0.01)	0.05 (0.11)	
IgM-RF, positive; Positive (-negative)	2 (−4)		13 (−1)	
ACPA, positive; positive (-negative)	1 (−5)		14	
Alkaline phosphatase; Unit/L (35–105)	89 (30)	66 (14)	71 (17)	
Fibrinogen; μmol/L (5.0–12)	14.5 (2.38)	9.1 0(0.8)		
Serum creatinine; units/L (45–90)	70 (16)	73.5 (15)	62 (7)	
Alanine transaminase; units/L (10–45)	21 (9)	24 (13)	18 (4)	
ANA positive; positive (-negative)	1 (−4)		1 (−11)	
Cell-free DNA; ng/mL	472.8 (60)	409.65 (51) *	356.7 (66)	317.3 (32)

^1^ All values are given with median and standard deviation in parentheses, and the reference values for healthy people are given where applicable. * The after-treatment samples of cfDNA only included eight patients.

## Data Availability

All raw and processed data are available through the PRIDE ProteomXchange repository under the number PXD016870 [57].

## Supplementary Materials

The following supporting information can be downloaded: https://www.mdpi.com/article/10.3390/jpm14050449/s1. Supplemental tables containing detailed lists of proteomics and immune-related measurements.
